# Dietary inflammatory index and risk of first myocardial infarction; a prospective population-based study

**DOI:** 10.1186/s12937-017-0243-8

**Published:** 2017-04-04

**Authors:** Stina Bodén, Maria Wennberg, Bethany Van Guelpen, Ingegerd Johansson, Bernt Lindahl, Jonas Andersson, Nitin Shivappa, James R. Hebert, Lena Maria Nilsson

**Affiliations:** 1 Department of Radiation Sciences, Oncology, Umeå University, Umeå, SE-901 87 Sweden; 2 Department of Public Health and Clinical Medicine, Nutritional Research, Umeå University, Umeå, SE-901 87 Sweden; 3 Department of Public Health and Clinical Medicine, Occupational and Environmental Medicine, Umeå University, Umeå, SE-901 87 Sweden; 4 Department of Public Health and Clinical Medicine, Research Unit Skellefteå, Umeå University, Umeå, SE-901 87 Sweden; 5 Cancer Prevention and Control Program, University of South Carolina, 915 Greene Street, Suite 241, Columbia, SC 29208 USA; 6 Department of Epidemiology and Biostatistics, Arnold School of Public Health, University of South Carolina, Columbia, SC 29208 USA; 7 Connecting Health Innovations LLC, 1417 Gregg St., Columbia, SC 29201 USA; 8 Arctic Research Centre (Arcum), Umeå University, Umeå, SE-901 87 Sweden

**Keywords:** DII Dietary inflammatory index, MI Myocardial infarction, NSHDS Northern Sweden health and disease study, VIP Västerbotten intervention programme, MONICA Monitoring of trends and determinants in cardiovascular disease, CVD cardiovascular disease, hsCRP high-sensitivity C-reactive protein, IL-6 interleukin 6

## Abstract

**Background:**

Chronic, low-grade inflammation is an established risk factor for cardiovascular disease. The inflammatory impact of diet can be reflected by concentrations of inflammatory markers in the bloodstream and the inflammatory potential of diet can be estimated by the dietary inflammatory index (DII^TM^), which has been associated with cardiovascular disease risk in some previous studies. We aimed to examine the association between the DII and the risk of first myocardial infarction (MI) in a population-based study with long follow-up.

**Method:**

We conducted a prospective case–control study of 1389 verified cases of first MI and 5555 matched controls nested within the population-based cohorts of the Northern Sweden Health and Disease Study (NSHDS), of which the largest is the ongoing Västerbotten Intervention Programme (VIP) with nearly 100 000 participants during the study period. Median follow-up from recruitment to MI diagnosis was 6.4 years (6.2 for men and 7.2 for women). DII scores were derived from a validated food frequency questionnaire (FFQ) administered in 1986–2006. Multivariable conditional logistic regression models were used to estimate odds ratios (OR) and 95% confidence intervals (CI), using quartile 1 (most anti-inflammatory diet) as the reference category. For validation, general linear models were used to estimate the association between the DII scores and two inflammatory markers, high-sensitivity C-reactive protein (hsCRP) and interleukin 6 (IL-6) in a subset (*n =* 605) of the study population.

**Results:**

Male participants with the most pro-inflammatory DII scores had an increased risk of MI [OR_Q4vsQ1_ = 1.57 (95% CI 1.21–2.02) *P*_trend_ = 0.02], which was essentially unchanged after adjustment for potential confounders, including cardiovascular risk factors [OR_Q4vsQ1_ = 1.50 (95% CI 1.14–1.99), *P*_trend_ = 0.10]. No association was found between DII and MI in women. An increase of one DII score unit was associated with 9% higher hsCRP (95% CI 0.03–0.14) and 6% higher IL-6 (95% CI 0.02–0.11) in 605 controls with biomarker data available.

**Conclusion:**

A pro-inflammatory diet was associated with an elevated risk of first myocardial infarction in men; whereas for women the relationship was null. Consideration of the inflammatory impact of diet could improve prevention of cardiovascular disease.

**Electronic supplementary material:**

The online version of this article (doi:10.1186/s12937-017-0243-8) contains supplementary material, which is available to authorized users.

## Background

Low-grade, systemic inflammation in the human body is associated with several chronic diseases and there is evidence that diet might affect the incidence of cardiovascular disease (CVD) through inflammatory mechanisms [1–5]. The impact of diet on chronic low-grade inflammation can be reflected by concentrations of inflammatory markers in the bloodstream, including cytokines, acute-phase proteins, soluble adhesion molecules and cytokine receptors [6]. A diet high in red meat, high-fat dairy, refined grains, processed meat, sweets, desserts and sugar-sweetened soft drinks has been associated with higher circulating levels of inflammatory markers in blood samples; whereas a food pattern high in fruits, vegetables and whole grains has been inversely related to inflammatory markers, such as C-reactive protein (CRP) and interleukin-6 (IL-6) [5–10]. Furthermore, a diet rich in fibre and low in fat and sugar was reported to be associated with lower systematic inflammation, which also has been suggested to have a protective role against CVD [11, 12].

The contribution of inflammation to the progression of atherosclerosis is well established, as is the association with CVD [13–17]. Atherosclerosis, characterized by accumulation of lipids, foam cell leucocytes, and fibrous elements in the large arteries, is associated with thrombosis, a complication responsible for myocardial infarction (MI) and most strokes. Multiple studies have confirmed that chronic low-grade inflammation causes an increased risk of CVD, and the risk of developing CVD rises sharply with age, particularly after long exposure to an unhealthy lifestyle involving tobacco use, excessive alcohol use, lack of physical activity, stress and consumption of a diet high in fat and red meat [1, 13, 14].

The literature-derived dietary inflammatory index (DII^TM^) has been designed to assess the inflammatory effect of diet, in any population and independent of specific dietary assessment method [18]. A pro-inflammatory diet, as estimated by the DII, has been associated with an increased risk of CVD in several populations, [19–23]; though some conflicting results also have been published [24, 25].

The aim of this study was to examine the DII scores in relation to the risk of first myocardial infarction (MI) in a prospective study with long follow-up.

## Methods

### Study design and population

We performed a nested case–control study within the Northern Sweden Health and Disease Study (NSHDS). Data were collected from two NSHDS cohorts, the Västerbotten Intervention Programme (VIP) and the Northern Sweden Monitoring Trends and Determinants in Cardiovascular Disease (MONICA) Study [26, 27]. The organization, sampling procedures, availability of samples/data, ethical considerations, and quality control program of the NSHDS are described in detail elsewhere [28]. The VIP is a long-term, ongoing programme including health interventions in the northern Swedish county of Västerbotten. All residents of Västerbotten, upon turning 40, 50 and 60 years of age (also 30 year olds during the period of 1985–1996 in all municipalities and continuously on a very small scale in some municipalities), are invited to a health screening at which they fill out a questionnaire addressing health history and lifestyle factors such as tobacco use, physical activity and diet. Participants are also asked to donate a blood sample for future research (stored in a biobank at −80 °C). A health examination is conducted at the screening event, including measurement of height and weight, blood pressure, blood fats and an oral glucose tolerance test as described previously [27]. The Northern Sweden MONICA Study follows essentially the same protocol as the VIP and includes 2000 or 2500 randomly selected 25–74 year olds (25–64 years in 1986) from the counties of Västerbotten and Norrbotten, stratified for age and sex and recruited approximately every 4–5 years since 1986. Mean participation rates for the time period of this study were approximately 60% for VIP and between 74 to 81% for the Northern Sweden MONICA Study. Minor selection bias has been reported for comparisons of participants and non-participants with respect to social characteristics [29]. In the present study, 89.3% of the participants were recruited through the VIP and 10.7% through the Northern Sweden MONICA Study.

The primary endpoint in this study was incidence of first definite MI (fatal and nonfatal, ICD-10 I21.0-I21.9). Cases occurring between January 1, 1986, and December 31, 2006, were identified through linkage between the NSHDS and the essentially complete and high-quality Northern Sweden MONICA Incidence Registry [30]. Exclusion criteria for cases recruited prior to 31 Dec 1999 were previous MI or stroke, or cancer other than non-melanoma skin cancer 5 years before or 1 year after MI. Exclusion criteria for cases recruited after 31 Dec 1999 were previous MI but previous stroke or cancer were not exclusion criteria.

**Fig. 1:**
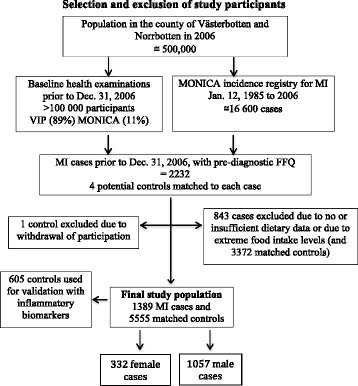
Flow chart illustrating the selection and exclusion of study participants. Abbreviations: VIP, Västerbotten Intervention Programme; MONICA, Monitoring Trends and Determinants in Cardiovascular Disease; MI, myocardial infarction; FFQ, food frequency questionnaire; FIL, food intake level

Figure 1 illustrates the selection and exclusion of study participants recruited from the VIP and the Northern Sweden MONICA cohort. Of 2232 eligible MI cases with food frequency questionnaires (FFQ), 843 cases were excluded due to lack of, or insufficient, dietary data, or due to extreme food intake levels (FIL) (lowest and highest 1% of the distribution as well as participants with energy intake >5000 kcal/day). Each case of first MI was matched with four control participants on sex, age (±3y), year of health examination (±3y), study cohort (VIP/MONICA), and FFQ version. Exclusion criteria for controls were the same as for cases. One control was excluded due to withdrawal of participation after the matching. Thus, the final study population involved 1389 MI-cases, including 332 women and 1057 men, and a total of 5555 matched controls. Median follow-up from recruitment to MI diagnosis was 6.4 years.

### Background variables

Tobacco smoking was classified as daily smokers, ex-smokers (former daily smokers) or never smokers (including occasional smokers and former occasional smokers). Total cholesterol concentrations (mmol/L) were measured in serum. Diabetes was defined as self-reported diabetes mellitus or diabetes mellitus diagnosed at the health examination (according to the WHO diagnostic criteria: fasting plasma glucose concentration ≥ 7.0 mmol/L or 2-h post-load plasma glucose concentration ≥ 11.1 mmol/L in MONICA project or ≥12.2 mmol/L in VIP, which used capillary blood). Education was dichotomized (post-secondary academic education, yes or no). Body mass index (BMI, kg/m^2^) and systolic blood pressure (mmHg) were based on measurements by health care professionals. Because questions about recreational physical activity varied in the two cohorts, three different variables concerning physical activity were harmonized on a three-level scale (low, medium, high) [31].

### Dietary assessment

Dietary data were collected at baseline using an optically readable, semi-quantitative FFQ, including 64 or 84 items, depending on year of measurement. Intake frequency had nine fixed alternatives (never, occasionally, 1–3 times/month, 1 time/week, 2–3 times/week, 4–6 times/week, 1 time/day, 2–3 times/day, ≥4 times/day). The frequency data, portion-size estimations and standard portion sizes were used to calculate daily intake of foods and nutrients. The FFQ has been validated and calibrated with repeated 24 h-recalls as well as biomarkers [32–34].

### Dietary inflammatory index (DII^TM^)

The development of the original DII and validations using inflammatory biomarkers have been presented elsewhere [18, 35, 36]. In brief, based on a comprehensive review of the scientific literature, 45 food parameters were identified as being related to one or more of a defined set of inflammatory markers (IL-1β, IL-4, IL-6, IL-10, TNF-α and C-reactive protein), and assigned an overall inflammatory effect score based on the strength of the association and the quality of the studies. For each parameter, food consumption data from eleven populations around the world were used to calculate global mean intakes and standard deviations. The global mean standard deviation was multiplied by the overall inflammatory effect score for each food parameter, and the sum of these food-parameter-specific contributions yields the final DII score. The DII score could range from −8.87 (maximally anti-inflammatory to +7.98 (maximally pro-inflammatory).

Intakes of 30 food parameters (of a total maximum of 45) could be obtained from our FFQ and were included in our DII, including total energy, total fat, saturated fat, trans fatty acids, cholesterol, monounsaturated fat, polyunsaturated fat, omega-6 fatty acids, omega-3 fatty acids, carbohydrates, protein, fibre, alcohol, caffeine, tea, folic acid, selenium, niacin, iron, zinc, thiamin, vitamin B_2,_ vitamin B_6_, vitamin B_12,_ vitamin A, vitamin E, vitamin C, vitamin D, magnesium and β-carotene (described with mean values and SD and compared with global mean intakes in the Additional file 1). The 15 food parameters excluded were eugenol, garlic, ginger, onion, saffron, turmeric, flanan-3-ol, flavones, flavonols, flavonones, anthocyanidins, isoflavones, pepper, thyme/oregano and rosemary. Caffeine intake was calculated as the sum of intake from coffee and tea. Tea in g/day was also kept as an independent parameter, as in the original DII.

### Validation of DII using circulating inflammatory markers

In addition to the main analyses, we validated DII scores using hsCRP and IL-6, which had previously been measured in a subset of the control subjects in this study (*n =* 605 of whom 81.8% were men) with recruitment dates between 1991–1999. Enzyme-linked immunosorbent assays (ELISA) were used (IMMULITE, Diagnostic Products Corporation, USA, for hsCRP and R & D Systems, Oxford, UK, for IL-6) [37]. The data were log-transformed and included as quartiles in general linear models, with the first DII quartile (most anti-inflammatory diet) as the reference category. β-coefficients for DII in quartiles and as continuous, as well as adjusted R^2^, were estimated in the general linear regression models. The first model was adjusted for total energy intake in kcal/day and for fasting duration prior to blood sample collection (classified to 0-4 h, 4–6 h, 6–8 h or >8 h). Fasting time is particularly relevant for IL-6 due to its shorter half-life (<2 h) and greater within-person variability compared to hsCRP [38]. The multivariable model was additionally adjusted for smoking, age, BMI, systolic blood pressure, diabetes, as well as the ratio between Apolipoprotein B and Apolipoprotein A1 (ApoB/ApoA1), which also was measured in these 605 controls.

### Statistical analyses

Sex-specific quartiles of DII were calculated based on the distribution of the controls. ANOVA tests for continuous variables and Chi-Square tests for categorical variables were used to assess differences in baseline characteristics across the DII quartiles. Odds ratios (OR) and 95% confidence intervals (CI) for the risk of first MI were estimated using conditional logistic regression models with the first DII quartile (Q1, most anti-inflammatory diet) as the reference category. An energy-adjusted model, adjusted for total energy intake in kcal/day was first constructed. To account for potential confounders, we constructed a multivariable model in which covariates were selected if they altered any of the risk estimates by more than 10%, or for theoretical importance as confounders. The final model included the covariates total energy intake, smoking, systolic blood pressure, total serum cholesterol, diabetes, education level, BMI and recreational physical activity. Because some of the covariates, particularly cholesterol, diabetes and BMI, are potential mediating factors of the association between DII and CVD, we also tested models excluding each of these in sensitivity tests. Participants with missing values for categorical covariates were included in the conditional logistic regressions using dummy categories. Participants with missing values for the continuous variables (systolic blood pressure and total serum cholesterol) were not included in the conditional logistic regression (in total, *n =* 127). Heterogeneity in risk estimates between sexes was tested by Chi-square analysis. In order to reduce the risk of reverse causation between inflammation and CVD a sensitivity test was also carried out, excluding cases with less than two years between recruitment and incidence of MI. We also stratified the data at the median lag time from recruitment to MI.

Interaction analyses were performed between DII and some potentially important effect modifiers (smoking, BMI and diabetes). We calculated the relative excess risk due to interaction (RERI) and the synergy index (S) [39]. 95% confidence intervals were determined using the approximate variance estimator [40]. We used the likelihood ratio tests of significance comparing models including versus those excluding the interaction term, in order to evaluate the significance of the multiplicative interaction.

All analyses were performed with SPSS® statistics version 23. All P values were two-tailed and P values ≤0.05 were considered to indicate statistical significance.

## Results

Table 1 presents baseline characteristics of the 5284 men and 1660 women according to sex-specific quartiles of DII. Missing values for background information, where present, are presented as frequencies. In this study, DII scores ranged from −4.16 (most anti-inflammatory) to +5.04 (most pro-inflammatory), with a mean in control subjects of 0.89 (1.66) in men and 1.08 (1.59) in women. Mean age at baseline was approximately 54 years across DII quartiles. Subjects with higher DII scores were characterized by lower education, higher smoking rates, lower physical activity and, in men, higher systolic blood pressure and higher serum total cholesterol concentrations, compared to subjects with lower DII scores. Diabetes appeared to be less common in subjects with higher DII in both men and women, though the differences did not reach statistical significance (data not shown). BMI did not vary by DII score in men, whereas obesity (BMI >30) was less common and overweight (BMI >25) more common in women with higher DII scores. The lag time from recruitment to MI was shorter in men than women, median 6.2 (25^th^, 75^th^ percentile: 3.5, 9.3) and 7.2 (4.4, 10.3) years, respectively, *P* = 0.001).

**Table 1:** Baseline characteristics of participants according to sex-specific quartiles of the dietary inflammatory index^a^

	Men (*n =* 5284)					Women (*n =* 1600)				
	Q1	Q2	Q3	Q4	*P* ^b^	Q1	Q2	Q3	Q4	*P* ^b^
n	1266	1319	1299	1400		407	422	404	427	
DII					<0.001					<0.001
Mean	–1.38	0.43	1.60	2.90		–1.14	0.69	1.81	2.93	
SD	0.83	0.37	0.33	0.53		0.87	0.38	0.29	0.47	
Min	–4.16	–0.25	1.05	2.16		–3.86	0.00	1.29	2.31	
Max	–0.25	1.05	2.16	5.04		0.00	1.29	2.30	4.31	
Age at baseline (Y)					0.079					0.331
Mean	53.4	54.1	53.9	54.0		54.9	54.3	54.7	54.0	
SD	7.47	7.19	7.36	7.38		7.68	8.17	7.40	8.28	
Postsecondary education (%)	20.6	18.9	13.8	12.9	<0.001	22.4	16.1	16.3	10.8	<0.001
Missing (n)	11	18	17	9		8	7	9	4	
Smoking status (%)					<0.001					<0.001
Daily smokers	15.4	16.8	22.6	28.8		15.7	22.3	22.0	34.2	
Ex-smokers	30.5	31.2	30.1	29.6		19.9	19.9	17.8	20.1	
Non-smokers	52.8	50.8	45.7	40.6		63.1	55.9	58.4	45.4	
Missing (n)	16	17	20	15		5	8	7	1	
Physical activity (%)^c^					<0.001					<0.001
Medium	47.7	45.6	44.5	37.7		54.8	43.4	42.9	40.5	
High	17.5	13.2	11.1	8.6		12.0	11.0	9.9	11.3	
Missing (n)	18	7	9	9		8	9	9	10	
BMI (kg/m^2^) (%)					0.944					0.017
<25	37.4	36.8	37.7	36.5		45.7	45.5	50.0	44.0	
25–29.9	51.1	50.3	49.9	50.7		32.9	35.3	36.9	40.7	
>30	11.5	12.9	12.3	12.8		21.4	19.2	13.1	15.2	
Missing (n)	1	1	3	0		0	0	0	0	
Diabetes (%)	8.2	7.5	6.2	5.9	0.054	7.1	8.1	5.4	4.9	0.211
Missing (n)	0	1	0	0		0	1	0	0	
Systolic BP (mmHg)					0.005					0.673
Mean	132.7	134.1	134.6	135.2		135.3	134.4	133.7	133.8	
SD	17.6	18.1	18.2	18.4		21.2	19.7	20.1	20.7	
Missing (n)	20	18	19	8		4	5	7	7	
S-cholesterol (mmol/L)					0.003					0.740
Mean	5.96	6.03	6.04	6.13		6.20	6.18	6.11	6.16	
SD	1.20	1.23	1.20	1.24		1.32	1.30	1.27	1.21	
Missing (n)	7	7	8	12		1	3	0	1	
Lag time (Y)^d^					0.498					0.142
Mean	6.80	6.81	6.41	6.43		6.88	7.79	8.14	6.96	
SD	4.05	3.87	4.00	4.08		3.88	3.96	4.17	4.26	

*Q* quartile, *DII* Dietary inflammatory index, *SD* Standard deviation, *Y* years, *BMI* Body mass index, *BP*, Blood pressure, *S-cholesterol,* Serum cholesterol, *Lag time* time from baseline to MI

^a^Quartile cutoffs for DII based on the controls

^b^P-values determined by ANOVA for continuous variables or Chi-Square tests for categorical variables

^c^Refers to recreational physical activity

^d^Years from health examination/recruitment to myocardial infarction

We observed statistically significant associations between the most pro-inflammatory DII scores (Q4) and both higher hsCRP and higher IL-6 in a subset of the control participants with biomarker data available (Additional file 2). In the multivariable model, an increase of one DII score unit was associated with 9% higher hsCRP (95% CI 0.03–0.14, *P*_trend_ = 0.003) and 6% higher IL-6 (95% CI 0.02–0.11, *P*_trend_ = 0.005). DII explained 1.7% of elevated hsCRP and 0.7% of elevated IL-6 concentrations in the energy-adjusted model (R^2^ adjusted 0.017 and 0.007 respectively).

Table 2 presents the risk associations between DII and MI. Men with the highest DII scores (Q4, most pro-inflammatory diet), had a higher risk of first MI compared to men with the lowest DII scores (Q1, most anti-inflammatory diet) (energy-adjusted OR_Q4vsQ1_ 1.57, 95% CI 1.22–2.02, *P*_trend_ = 0.02). This finding was essentially unchanged by adjusting for potential confounders, though the linear trend, using DII score as a continuous variable, lost statistical significance (multivariable OR_Q4vsQ1_ 1.50, 1.14–1.99, *P*_trend_ = 0.10). Excluding cholesterol, BMI or diabetes, potential mediating factors, from the multivariable model had negligible effects on the risk estimates (Additional file 3). Excluding cases with less than two years between baseline and MI diagnosis did not affect the magnitude or statistical significance of the risk estimates (multivariable OR_Q4vsQ1_ for men 1.56, 95% CI 1.16–2.10, *P*_trend_ = 0.10, Additional file 3). Stratifying at the median lag time of 6.2 years for men and 7.2 years for women also yielded largely consistent results (Additional file 3). We did not find any association between DII and MI risk in women, and the Chi-square test for heterogeneity of results between sexes was statistically significant (*P* = 0.04).

**Table 2:** The association between the dietary inflammatory index and first myocardial infarction for men and women^a^

	Q1	Q2		Q3		Q4		P trend^b^
Men								
Cases/controls (*n*)	210/1056	261/1058		242/1057		344/1056		
Range DII score	–4.16 – -0.25	–0.25 – 1.05		1.05 – 2.16		2.16 – 4.72		
	OR	OR	95% CI	OR	95% CI	OR	95% CI	
Energy-adjusted^c^	ref	1.22	0.98–1.51	1.12	0.88–1.42	1.57	1.21–2.02	0.018
Multivariable^d^	ref	1.20	0.95–1.53	1.13	0.87–1.45	1.50	1.14–1.99	0.097
Women								
Cases/controls (*n*)	75/332	90/332		72/332		95/332		
Range DII score	–3.86 – 0.00	0.00 – 1.29		1.29 – 2.31		2.31 – 4.31		
	OR	OR	95% CI	OR	95% CI	OR	95% CI	
Energy-adjusted^c^	ref	1.09	0.75–1.58	0.82	0.54–1.23	1.01	0.65–1.56	0.423
Multivariable^d^	ref	1.05	0.69–1.59	0.82	0.51–1.30	0.83	0.50–1.36	0.088

*Q* quartile of DII, *DII* Dietary inflammatory index, *ref* reference

^a^Conditional logistic regression models presented with odds ratio (OR) and 95% confidence interval (CI)

^b^ *P* for trend when DII used as a continuous variable

^c^Adjusted for total energy intake in kcal/day

^d^Adjusted for total energy intake, body mass index, physical activity, systolic blood pressure, total serum cholesterol, diabetes, smoking, and postsecondary academic education

**Fig. 2:**
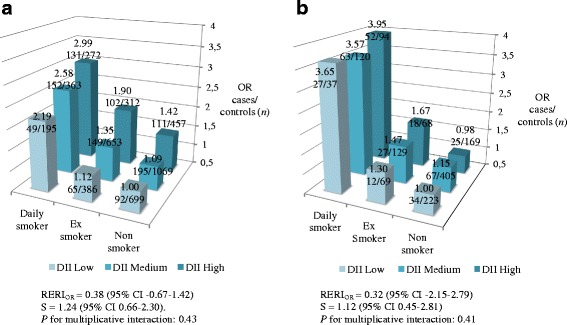
Abbreviations: RERI, relative excess risk due to interaction; S, synergy index. Joint effect of dietary inflammatory index (DII) and smoking status, on the risk of first myocardial infarction (MI) in **a** men **b** women. Odds ratios (OR) were estimated from multiple regression models adjusted for total energy intake, total serum cholesterol, systolic blood pressure, body mass index (BMI), diabetes, and postsecondary academic education. The calculations of RERI and S were performed comparing daily smokers/non-smokers and low DII/high DII

Multivariable interaction models for DII and smoking, BMI, and diabetes in relation to MI risk are presented as 3D bar graphs in Fig. 2 and Additional file 4. A positive additive interaction was found for DII and smoking in men, although the RERI and S were not statistically significant (Fig. 2a, RERI_OR_ = 0.38 (95% CI −0.67–1.42), S = 1.24 (0.66–2.30). There was no interaction on the multiplicative scale (*P* = 0.43). In women, a small positive additive interaction was seen for DII and smoking (non-significant, Fig. 2b), Neither BMI nor diabetes interacted with DII in relation to MI risk in men or women (Additional file 4).

## Discussion

In this population-based, nested case–control study, men with the highest DII scores, representing the most pro-inflammatory diet, had a higher risk of first MI. In women, no association was found. We also observed a positive, but non-significant, additive interaction between the DII and smoking in relation to MI risk in both men and women, underscoring the potential importance of evaluating pro-inflammatory lifestyle behaviors as effect modifiers of the association between DII and inflammation-related disease.

Our findings are consistent with some previous results for DII and incident CVD as well as all-cause mortality [19–23]; although null associations have also been reported [24, 25]. Effect sizes for CVD risk have generally been in the range of approximately OR 1.5 to 2, which is comparable to our results for men. The DII scores in our study population were also comparable to those of previous studies [19–22, 36], including the SEASONS study, in which DII was validated against the inflammatory marker hsCRP [36]. Also in line with previous studies [35, 36], the DII was weakly, but statistically significantly, associated with both hsCRP and the inflammatory marker IL-6 in a subset of our control participants with biomarker data available. The DII based on our FFQ and applied to our study population appears to be sufficient for this type of investigation, and for interpretation of the results in relation to previous findings.

Our finding of a significant sex difference in the association between DII and MI risk was not entirely without precedent. Null results have been reported for women in other studies of DII and CVD [21, 25], though in one of the studies the female cases were few and relatively young (mean age 35.4 years) [21]. Our study included a lower number of women than men (332 and 1057 cases, respectively), which reflects the lower incidence rate and higher age at first MI in women. Although this may explain the lack of association for women in our study, the sample size should be sufficient to detect an association. Furthermore, the OR and not just the CI and P-values, are consistent with a null association. The importance of investigating sex-related differences regarding diet and CVD risk profile has been emphasized in studies of the Mediterranean diet [41, 42]. In a 12-week dietary intervention study, men had a pronounced beneficial change in long-term, post-intervention dietary intake compared to women, as well as a greater improvement in metabolic profile [42].

Dietary changes in the northern Swedish population occurred over the study period, which might be speculated to influence our analyses. The VIP was initiated largely as a response to high CVD mortality rates in the county of Västerbotten in the mid-1980s [26, 27]. It is a strategic, population-wide programme involving both general initiatives, such as a healthy choice “key hole” label on foods, and individual health screening and lifestyle counseling. An impact of the VIP on all-cause and cardiovascular mortality has been reported [43]. However, a low-carbohydrate/high-fat dietary trend also occurred in Sweden toward the end of our study period [44]. Total fat intake (contributing essentially to a more pro-inflammatory DII score) began to increase after 2004 in both sexes, but more so in women, after decreasing during the first seven years of the VIP (1986–1992), and total serum cholesterol concentrations, after decreasing for several years, leveled off in 2004–2007 and then began to rise [45]. Although such temporal changes were probably reflected in the DII, the use of a matched case–control rather than cohort design for our study minimized the potential bias related to changes in dietary habits in the population during the data collection period.

Underreporting of dietary intake is a known problem in nutritional epidemiology. In a validation of our FFQ using repeated 24-h recalls, the prevalence and magnitude of underreporting of energy intake were directly related to BMI [46], and especially foods characterized as socially undesirable (e.g., high-fat foods and simple carbohydrates) were underreported [47, 48]. This may explain why BMI did not demonstrate an expected direct relationship with DII, and emphasizes the importance of adjusting for BMI and energy intake in studies using dietary data. Underreporting is most likely to lead to underestimation of diet-disease relations, and the null finding in women could be explained by sex-differential social desirability bias that is much more pronounced in women [48–50].

BMI has the potential to act as a mediator between diet, low-grade chronic inflammation and inflammation-related diseases, and not just as a confounder. Body fatness creates a pro-inflammatory metabolic environment, and BMI is positively related to CRP and other inflammatory markers [3, 4]. For this reason, as for the other potential mediating factors, total serum cholesterol concentrations and diabetes, we tested sequential exclusion from the multivariable model with no material effects on risk estimates. Although we excluded subjects with extreme energy intakes, underreporting is still an issue in self-reported FFQs.

Diabetes did not demonstrate the expected direct relationship with a more pro-inflammatory diet, (higher DII scores). Rather, an opposite trend was observed, though it did not reach statistical significance. This is not consistent with the causal chain, in which low-grade inflammation contributes to the development of metabolic diseases. Participants with diabetes may, speculatively, have changed their dietary patterns after advice from health care professionals.

The effect sizes observed for the DII in relation to cardiovascular endpoints are generally modest [19–23], which is not surprising, given the many other potential contributing factors to low-grade chronic inflammation. However, the general consistency of results suggests that the inflammatory impact of diet is a potential target for CVD prevention. Also, an anti-inflammatory diet, characterized by a low intake of saturated fat, high intake of vegetables and fibre, is beneficial for preventing CVD through mechanisms other than inflammation, such as lowering blood lipids and thus reducing the risk of thrombosis.

The main strengths of this study include its prospective design, which minimizes recall bias and reverse causation, and the population-based nature of the study cohorts. The sample size was large (*n =* 6944, including 1389 cases), with enough statistical power for subgroup and interaction analyses. However, there are also limitations. We were only able to include 30 of the 45 food parameters in the original DII. The 15 parameters excluded were all anti-inflammatory, which may have contributed to more pro-inflammatory DII scores. Other studies investigating the DII in relation to cardiovascular disease had similar exclusions of food parameters characterized as anti-inflammatory [21, 23]. The semi-quantitative FFQ has inherent weaknesses, particularly underreporting, but it has demonstrated validity similar to other FFQs [32–34]. Our analyses were based on a single health-screening occasion per participant, and we can, therefore, not account for intra-individual changes in dietary and lifestyle habits. Although we lacked data on aspirin use, we were able to adjust for a number of important cardiovascular risk factors. As always with observational studies, we cannot answer the question of causality, and thus a causal relationship between a predictor and the outcome cannot be confirmed.

## Conclusions

In this prospective, population-based study with long follow-up, a more pro-inflammatory diet as estimated by the DII was associated with an increased risk of first MI in men. These findings support inflammation as a link between diet and cardiovascular risk as well as the usefulness of the DII for estimating the inflammatory impact of diet.

## Additional files

Additional file 1: Food parameters included in the NSHDS-DII. (DOCX 113 kb)

Additional file 2: Associations between Dietary inflammatory index and the inflammatory biomarkers C-reactive protein and Interleukin 6. (DOCX 92 kb)

Additional file 3: Sensitivity and subgroup analyses for the association between the dietary inflammatory index and myocardial infarction. (DOCX 136 kb)

Additional file 4: Statistical interactions with DII-BMI and DII-diabetes. (PDF 786 kb)
