# Supplementary material for: Dietary inflammatory index and risk of first myocardial infarction; a prospective population-based study
Source: Nutr J. 2017 Apr 4;16:21. doi: 10.1186/s12937-017-0243-8 (PMC5379659; doi:10.1186/s12937-017-0243-8)
Supplement: Supplementary file 1 — Food parameters included in the NSHDS-DII. (DOCX 113 kb) [file 12937_2017_243_MOESM1_ESM.docx]

|  | OIES^b^ | GDI^c^ | | Men  n=5284 | | Women  n=1600 | |
| --- | --- | --- | --- | --- | --- | --- | --- |
|  |  | Mean | SD | Mean | SD | Mean | SD |
| Saturated fat (g/day) | 0.373 | 28.6 | 8.0 | 34.0 | 13.3 | 21.4 | 8.42 |
| Total fat (g/day) | 0.298 | 71.4 | 19.4 | 78.8 | 27.9 | 50.1 | 18.0 |
| Trans fatty acids (g/day) | 0.229 | 3.15 | 3.75 | 2.81 | 1.51 | 1.80 | 0.92 |
| Energy (kcal/day) | 0.180 | 2056 | 338 | 2036 | 605 | 1441 | 426 |
| Cholesterol (mg/day) | 0.110 | 279.4 | 51.2 | 237.2 | 91.3 | 159.8 | 62.2 |
| Vitamin B_12_ (µg/day) | 0.106 | 5.15 | 2.70 | 5.21 | 2.59 | 4.16 | 1.89 |
| Carbohydrates (g/day) | 0.097 | 272.2 | 40.0 | 250.0 | 82.5 | 188.2 | 59.2 |
| Iron (mg/day) | 0.032 | 13.35 | 3.71 | 15.0 | 5.42 | 11.15 | 3.99 |
| Protein (g/day) | 0.021 | 79.4 | 13.9 | 71.2 | 22.9 | 53.8 | 17.1 |
| MUFA (g/day) | -0.009 | 27.0 | 6.1 | 26.0 | 9.48 | 16.8 | 5.93 |
| Vitamin B_2_ (mg/day) | -0.068 | 1.70 | 0.79 | 1.54 | 0.56 | 1.26 | 0.43 |
| Tiamin (mg/day) | -0.098 | 1.70 | 0.66 | 1.30 | 0.43 | 1.13 | 0.40 |
| Coffein (mg/day) | -0.110 | 80.5 | 66.7 | 224 | 97.8 | 223 | 97.6 |
| n-6 fatty acids (g/day) | -0.159 | 10.8 | 7.50 | 7.87 | 3.88 | 5.30 | 2.51 |
| Folic acid (µg/day) | -0.190 | 273 | 70.7 | 232 | 82.2 | 218 | 88.4 |
| Selenium (µg/day) | -0.191 | 67.0 | 25.1 | 24.3 | 9.08 | 19.6 | 7.16 |
| Niacin (mg/day) | -0.246 | 25.9 | 11.8 | 16.0 | 5.60 | 12.6 | 4.08 |
| Alcohol (g/day) | -0.278 | 13.98 | 3.72 | 5.15 | 5.55 | 1.84 | 2.63 |
| Zink (mg/day) | -0.313 | 9.84 | 2.19 | 8.85 | 2.99 | 7.36 | 2.43 |
| PUFA (g/day) | -0.337 | 13.9 | 3.76 | 11.0 | 4.99 | 7.30 | 3.19 |
| Vitamin B_6_ (mg/day) | -0.365 | 1.47 | 0.74 | 2.06 | 0.75 | 1.77 | 0.60 |
| Vitamin A (mg/day) | -0.401 | 983.9 | 518.6 | 909.0 | 503.5 | 615.5 | 334.7 |
| Vitamin E (mg/day) | -0.419 | 8.73 | 1.49 | 6.88 | 2.68 | 5.37 | 1.99 |
| Vitamin C (mg/day) | -0.424 | 118.2 | 43.46 | 81.9 | 48.9 | 83.1 | 50.7 |
| n-3 fatty acids (g/day) | -0.436 | 1.06 | 1.06 | 1.93 | 0.80 | 1.29 | 0.51 |
| Vitamin D (µg/day) | -0.446 | 6.26 | 2.21 | 6.01 | 2.46 | 4.13 | 1.61 |
| Magnesium (mg/day) | -0.484 | 310.1 | 139.4 | 317.1 | 96.9 | 269.1 | 78.4 |
| Tea (g/day) | -0.536 | 1.69 | 1.53 | 0.54 | 0.75 | 0.56 | 0.76 |
| β-Carotene (µg/dag) | -0.584 | 3718 | 1720 | 2756 | 2972 | 4690 | 4644 |
| Fibre (g) | -0.663 | 18.8 | 4.9 | 21.3 | 8.11 | 17.9 | 6.76 |

Supplementary Table 1

Food parameters (n=30) included in the dietary inflammatory index (DII) in the Northern Sweden Health and Disease Study (NSHDS)

Abbreviations: OIES, overall inflammatory effect score; GDI, Global daily intake; MUFA, monounsaturated fat; n-6, omega-6; PUFA, polyunsaturated fat; n-3, omega-3

^a^ Ranked from the highest score [most pro-inflammatory (saturated fat)] to the lowest score [most anti-inflammatory (Fibre)]

^b^ According to the DII ranked from the most pro-inflammatory to the most anti-inflammatory parameter [18]

^c^ According to the mean intake of eleven populations around the world from where the original DII was calculated [18]
