# Supplementary material for: Dietary inflammatory index and risk of first myocardial infarction; a prospective population-based study
Source: Nutr J. 2017 Apr 4;16:21. doi: 10.1186/s12937-017-0243-8 (PMC5379659; doi:10.1186/s12937-017-0243-8)
Supplement: Supplementary file 2 — Associations between Dietary inflammatory index and the inflammatory biomarkers C-reactive protein and Interleukin 6. (DOCX 92 kb) [file 12937_2017_243_MOESM2_ESM.docx]

Supplementary Table 2

The association between the dietary inflammatory index and two inflammatory biomarkers (hsCRP and IL6) in 605 control subjects (81.8% men)

|  | Energy-adjusted^a^ | | | *P* trend | Multivariable^b^ | | | *P* trend |
| --- | --- | --- | --- | --- | --- | --- | --- | --- |
| Log-hsCRP^c^ | *n* | β-coefficient | 95% CI |  | *n* | β-coefficient | 95% CI |  |
| DII score^d^ |  |  |  |  |  |  |  |  |
| Q1 | 144 | ref | - |  | 139 | ref | - |  |
| Q2 | 137 | 0.08 | -0.15-0.30 |  | 127 | 0.11 | -0.09-0.32 |  |
| Q3 | 142 | 0.26 | 0.02-0.50 |  | 134 | 0.25 | 0.02-0.47 |  |
| Q4 | 142 | 0.35 | 0.07-0.62 |  | 138 | 0.41 | 0.16-0.67 |  |
| continuous | 565 | 0.07 | 0.01-0.13 | 0.022 | 538 | 0.09 | 0.03-0.14 | 0.003 |
| *R^2^ (adjusted)* |  | 0.017 |  |  |  | 0.216 |  |  |
|  |  |  |  |  |  |  |  |  |
| Log-IL6 |  |  |  |  |  |  |  |  |
| DII score^d^ |  |  |  |  |  |  |  |  |
| Q1 | 147 | ref | - |  | 142 | ref | - |  |
| Q2 | 142 | 0.12 | -0.05-0.28 |  | 132 | 0.14 | -0.02-0.29 |  |
| Q3 | 148 | 0.19 | 0.01-0.37 |  | 140 | 0.16 | -0.01-0.33 |  |
| Q4 | 147 | 0.29 | 0.09-0.50 |  | 143 | 0.26 | 0.06-0.46 |  |
| continuous | 584 | 0.06 | 0.01-0.10 | 0.014 | 557 | 0.06 | 0.02-0.11 | 0.005 |
| *R^2^ (adjusted)* |  | 0.007 |  |  |  | 0.108 |  |  |

Abbreviations: Log-hsCRP, log-transformed high-sensitivity C-reactive protein; Log-IL6, log-transformed interleukin-6; CI, confidence interval; DII, dietary inflammatory index; Q, quartile of DII

^a^Adjusted for total energy intake in kcal/day and for fasting time before blood sample draw (and classified to 0-4h, 4-6 h, 6-8 h or >8h)

^b^Adjusted for energy intake, fasting time, smoking, age, Apolipoprotein B/ApolipoproteinA1, systolic blood pressure, diabetes, and body mass index

^c^Individuals with hsCRP level of >10mg/L (n=22) were excluded since values that high could be caused by acute inflammation

^d^Quartile cutoffs: Q1 (most anti-inflammatory): -4.56- -0.54, Q2: - 0.55- 0.84, Q3: 0.85- - 2.04, Q4 (most pro-inflammatory): 2.05- 4.02
