# Supplementary material for: Dietary inflammatory index and risk of first myocardial infarction; a prospective population-based study
Source: Nutr J. 2017 Apr 4;16:21. doi: 10.1186/s12937-017-0243-8 (PMC5379659; doi:10.1186/s12937-017-0243-8)
Supplement: Supplementary file 3 — Sensitivity and subgroup analyses for the association between the dietary inflammatory index and myocardial infarction. (DOCX 136 kb) [file 12937_2017_243_MOESM3_ESM.docx]

Supplementary Table 3

Sensitivity and subgroup analyses for the association between the dietary inflammatory index and myocardial infarction

|  | Q1 | Q2 | | Q3 | | Q4 | | *P* trend^a^ |
| --- | --- | --- | --- | --- | --- | --- | --- | --- |
|  | OR | OR | 95 % CI | OR | 95 % CI | OR | 95 % CI |  |
| Men |  |  | |  | |  | |  |
| Multivariable^b^ | ref | 1.19 | 0.95-1.53 | 1.13 | 0.87-1.45 | 1.50 | 1.14-1.99 | 0.097 |
| Excluding S-cholesterol | ref | 1.24 | 0.99-1.56 | 1.16 | 0.90-1.48 | 1.56 | 1.18-2.05 | 0.037 |
| Excluding BMI | ref | 1.18 | 0.94-1.49 | 1.09 | 0.85-1.41 | 1.46 | 1.11-1.92 | 0.125 |
| Excluding diabetes | ref | 1.17 | 0.93-1.48 | 1.07 | 0.83-1.38 | 1.40 | 1.07-1.85 | 0.265 |
| Excluding cases <2Y to MI | ref | 1.22 | 0.95-1.56 | 1.10 | 0.83-1.45 | 1.56 | 1.16-2.10 | 0.095 |
| If <6.2 Y to MI^c^ | ref | 1.20 | 0.85-1.68 | 1.15 | 0.80-1.65 | 1.48 | 1.00-2.20 | 0.313 |
| If >6.2 Y to MI^c^ | ref | 1.21 | 0.88-1.67 | 1.07 | 0.74-1.54 | 1.55 | 1.04-2.30 | 0.184 |
| Women |  |  |  |  |  |  |  |  |
| Multivariable^b^ | ref | 1.05 | 0.69-1.59 | 0.82 | 0.51-1.30 | 0.83 | 0.50-1.35 | 0.088 |
| Excluding S-cholesterol | ref | 1.08 | 0.72-1.62 | 0.85 | 0.54-1.33 | 0.91 | 0.56-1.46 | 0.139 |
| Excluding BMI | ref | 1.06 | 0.70-1.60 | 0.84 | 0.53-1.32 | 0.88 | 0.54-1.42 | 0.099 |
| Excluding diabetes | ref | 1.03 | 0.69-1.55 | 0.78 | 0.50-1.23 | 0.77 | 0.48-1.30 | 0.030 |
| Excluding cases <2Y to MI | ref | 1.07 | 0.69-1.65 | 0.77 | 0.48-1.25 | 0.75 | 0.45-1.26 | 0.039 |
| If <7.2 Y to MI^c^ | ref | 0.88 | 0.49-1.59 | 0.60 | 0.30-1.21 | 0.92 | 0.45-1.89 | 0.305 |
| If >7.2 Y to MI^c^ | ref | 1.18 | 0.65-2.14 | 0.99 | 0.53-1.86 | 0.68 | 0.33-1.39 | 0.169 |

Abbreviations: Q, quartile of DII; S-cholesterol, serum-cholesterol; BMI, body mass index; Y, years; MI, myocardial infarction; DII, dietary inflammatory index

^a^Calculated using DII score as a continuous variable in the multivariable model

^b^As presented in Table 2. Adjusted for energy intake, body mass index, physical activity, systolic blood pressure, total serum cholesterol, diabetes, smoking, and postsecondary academic education

^c^Adjusted according to the full multivariable model
