# Supplementary material for: Dietary inflammatory index and risk of first myocardial infarction; a prospective population-based study
Source: Nutr J. 2017 Apr 4;16:21. doi: 10.1186/s12937-017-0243-8 (PMC5379659; doi:10.1186/s12937-017-0243-8)
Supplement: Supplementary file 4 — Statistical interactions with DII-BMI and DII-diabetes. (PDF 786 kb) [file 12937_2017_243_MOESM4_ESM.pdf]

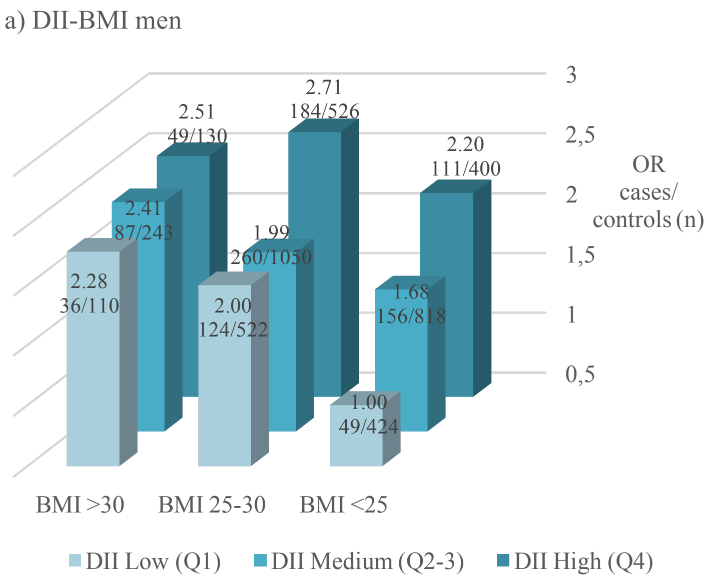

$RERI_{OR}$ = -0.87 (95% CI -2.28-0.55)
S = 0.62 (95% CI 0.29-1.29),
P for multiplicative interaction: 0.11

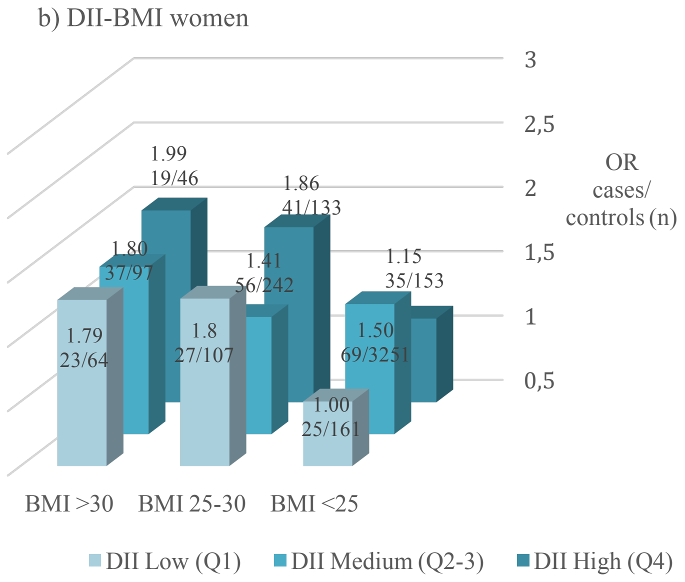

$RERI_{OR}$ = 0.05 (95% CI: -1.60-1.71)
S = 1.05 (95% CI: 0.19-6.04)
P for multiplicative interaction: 0.44

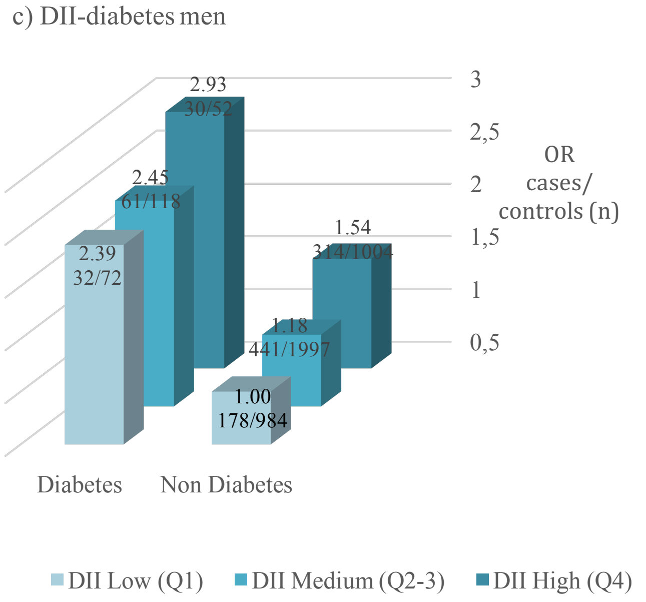

$RERI_{OR}$ = 0.0 (95% CI: -1.82-1.82)
S = 1.0 (95% CI: 0.39-2.57)
P for multiplicative interaction:0.25

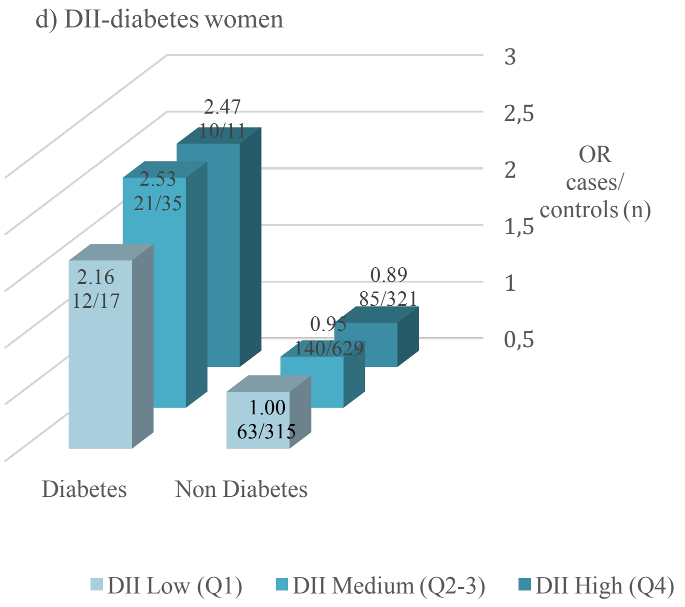

$RERI_{OR}$ = 0.42 (95% CI: -2.57-3.40)
S = 1.4 (95% CI: 0.13-14.7)
P for multiplicative interaction: 0.72

Abbrevations: RERI, relative excess risk due to interaction; S, synergy index; DII, dietary inflammatory index; BMI, body mass index; MI, myocardial infarction

Supplementary Figure 1. Joint effect of DII and BMI on the risk of first myocardial infarction (MI) in a) men and b) women, and joint effect of DII and diabetes on the risk of MI in c) men and d) women. Odds ratios (OR) were estimated from multiple regression models adjusted for total energy intake, total serum cholesterol, systolic blood pressure, smoking status, postsecondary academic education, physical activity diabetes (a and b), and BMI (c and d). The calculations of *RERI* and *S* were performed comparing BMI <25/BMI >30 and low DII/high DII for figure 1a and b.
